# Breaking (Light) Barriers: Photoacoustics in Atherosclerotic Diseases

**DOI:** 10.1111/jcmm.70933

**Published:** 2025-10-31

**Authors:** Kayleigh van Dijk, Margreet R. de Vries

**Affiliations:** ^1^ Einthoven Laboratory for Experimental Vascular Medicine, Department of Surgery LUMC Leiden the Netherlands; ^2^ Department of Surgery and the Heart and Vascular Center Brigham & Women's Hospital and Harvard Medical School Boston Massachusetts USA

**Keywords:** atherosclerosis, cardiovascular disease, nanoparticles, photoacoustic imaging

## Abstract

Photoacoustic imaging (PAI) is an emerging imaging modality that provides high‐resolution, spatiotemporal insights into anatomical structures, functional parameters and molecular characteristics of tissues. By leveraging both endogenous absorbers, such as lipids and collagen, and exogenous contrast agents like nanoparticles and molecular dyes, PAI offers a comprehensive approach to characterising atherosclerosis. This review describes the technical principles underlying various PAI modalities and spectral unmixing techniques. Furthermore, it addresses the application of endogenous PAI for assessing atherosclerotic plaque stability and the utility of exogenous contrast. We examine the current landscape of PAI in cardiovascular research, its strengths in evaluating plaque vulnerability and the challenges and future directions for its clinical translation.

## 1 Introduction

**FIGURE 1:**
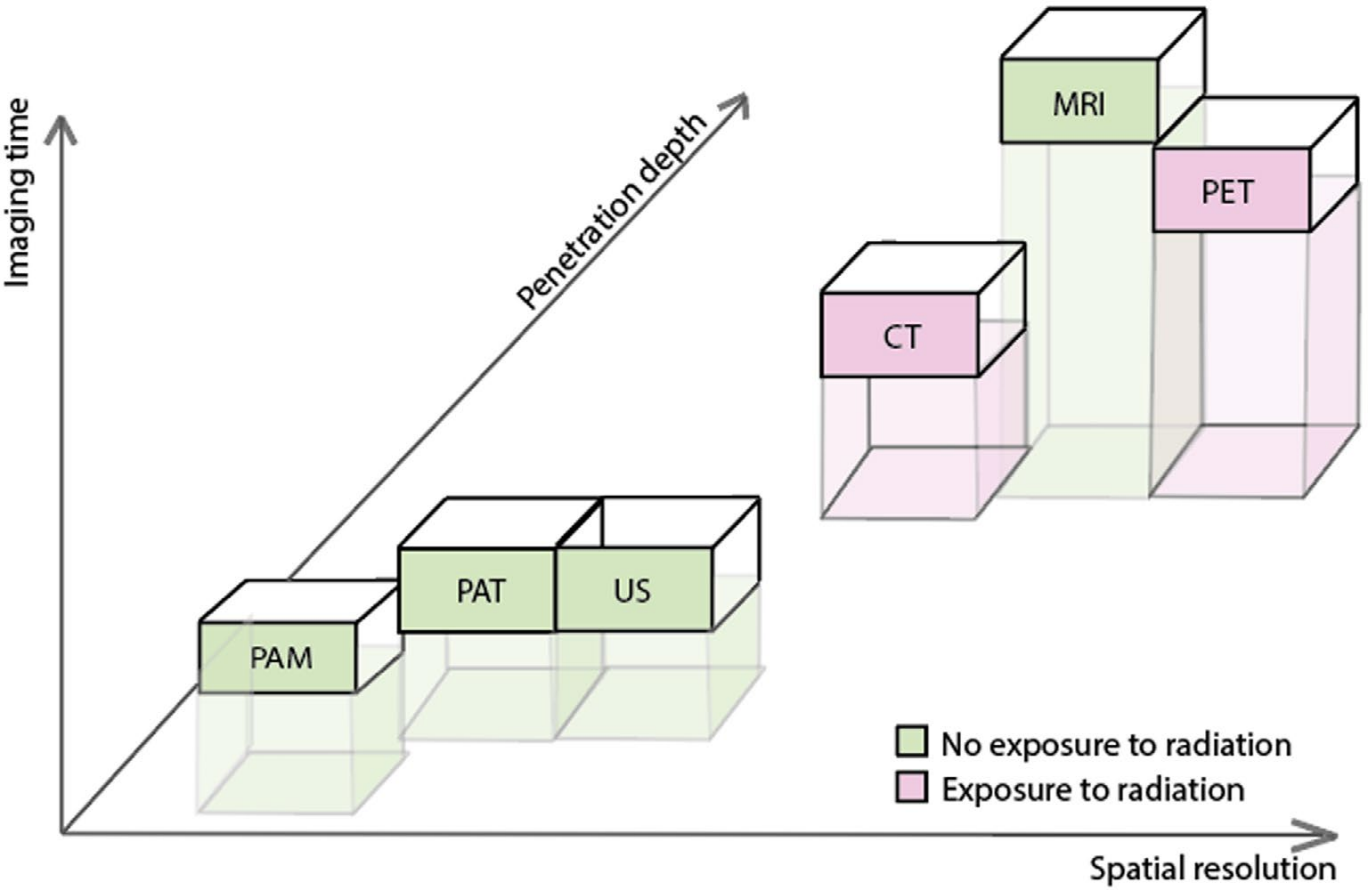
Photoacoustic imaging methods in reference to frequently used cardiovascular imaging strategies. From left to right: photoacoustic microscopy (PAM), photoacoustic tomography (PAT), ultrasound (US), computed tomography (CT), magnetic resonance imaging (MRI), positron emission tomography (PET).

Cardiovascular diseases (CVDs) remain the leading cause of mortality worldwide. This underscores the importance of advanced, noninvasive imaging techniques for early detection and disease monitoring [1, 2]. Widely adopted modalities such as ultrasound (US), magnetic resonance imaging (MRI), computed tomography (CT) and positron emission tomography (PET) play a central role in evaluating cardiovascular structure and function (Figure 1). Photoacoustic imaging (PAI) uniquely couples high optical contrast with deep tissue penetration and fine spatial resolution. PAI is often combined with US to merge molecular and structural imaging, offering complementary insights that enhance and expand current cardiovascular imaging approaches.

PAI operates on the ‘light in, sound out’ principle. In short, pulsed laser light is absorbed by endogenous chromophores, causing thermoelastic expansion resulting in acoustic waves, which can be detected by US transducers [3]. The significantly lower acoustic scattering compared to light scattering enables PAI to penetrate deeper than pure optical imaging. Endogenous markers, such as haemoglobin, lipids and water, have unique spectra that can be distinguished in the near‐infrared (NIR) window (700–2500 nm), the primary operating range of PAI [4].

PAI can be implemented in different configurations depending on the imaging depth and spatial resolution required, making it adaptable to a range of cardiovascular applications. Photoacoustic microscopy (PAM) [5] provides cellular‐level resolution for shallow structures like skin microvasculature, while photoacoustic tomography (PAT) [6] images deeper tissues like larger vessels and plaques. As illustrated in Figure 1, PAM and PAT complement established cardiovascular imaging modalities by offering high spatial resolution, rapid acquisition times and minimal invasiveness. Compared to PET and MRI, PAI offers shorter acquisition times without ionising radiation. Although limited in penetration depth compared to MRI or CT, PAI's spatial resolution can reach near single‐cell levels, making molecular imaging possible. Key factors such as safety, cost, portability and ease of use also contribute to the increasing appeal of PAI for longitudinal monitoring in both clinical and preclinical studies of cardiovascular disease.

Building on its established application in oncology, dermatology and neuroscience, PAI is gaining increasing attention for cardiovascular applications. Its ability to provide high‐resolution, depth‐adaptable imaging in a minimally invasive manner offers significant advantages for in vivo studies of vascular pathology across both superficial and deep tissue compartments. This review discusses recent advances in the application of PAI to cardiovascular disease, with a focus on imaging both intrinsic tissue components and extrinsic contrast agents. Emphasis is placed on the detection of features associated with vulnerable atherosclerotic plaques, as well as current limitations and prospective avenues for clinical translation.

## 2 Photoacoustic Imaging Modalities

PAI can be tailored to different cardiovascular applications through specific system designs that determine resolution, penetration depth and field of view. The two most common implementations, PAM and PAT, differ in their optical and acoustic focusing strategies. In addition to these imaging configurations, intravascular photoacoustic imaging (IVPAI) has emerged as a powerful modality for high‐resolution imaging of atherosclerotic lesions from within the lumen.

PAM is preferred when high spatial resolution is required. This is achieved by tightly focusing light onto the tissue, which allows for detailed imaging at the microscopic level [5]. PAM can be further divided into two types: optical‐resolution PAM (OR‐PAM), where the resolution is determined by how finely the light is focused, and acoustic‐resolution PAM (AR‐PAM), where the focus is based on acoustic waves. OR‐PAM can resolve features as small as a few microns but is limited in imaging depths, typically < 1 mm. AR‐PAM, on the other hand, sacrifices some resolution but can reach several millimetres into tissue, making it better suited for imaging structures such as skin. These PAM configurations are particularly useful for monitoring cutaneous microvessels in patients with peripheral artery disease [7, 8].

For imaging larger structures or deeper tissue layers, PAT is more appropriate. Unlike PAM, PAT utilises a wide beam to illuminate a broader area [3, 6, 9]. The resulting US signals are captured by detectors arranged around the tissue, often as part of a handheld probe or ring‐shaped array [3, 10]. PAT systems can image several centimetres into tissue with spatial resolution in the sub‐millimetre range, making systems using PAT suitable for visualising entire plaques and surrounding vasculature. This approach has been successfully used in both animal models and early‐stage human studies. Because PAT balances depth, resolution and scalability, it serves as the foundation for many translational and clinical applications of PAI in cardiovascular disease. For example, currently, a clinical trial is ongoing using PAT for the identification of lower extremity artery disease (NCT06579326).

Building on these non‐invasive approaches, IVPAI brings PAI directly into blood vessels. IVPAI can produce high‐resolution, cross‐sectional images of plaque components from within the artery using catheter‐based probes that combine laser delivery and US detection [11, 12]. By imaging from inside the vessel, IVPAI limits light scattering through tissue, enabling accurate visualisation of features located several millimetres deep in the vessel wall. This makes it especially effective for imaging plaques. Although still under development for routine clinical use, IVPAI has demonstrated significant potential.

## 3 Spectral Photoacoustic Imaging

Across all configurations of PAI, the most compelling strength is the ability to distinguish between different tissue components based on their unique optical absorption profiles. Endogenous chromophores such as oxyhaemoglobin, deoxyhaemoglobin, lipids and collagen have distinct absorption spectra, allowing them to be identified by illuminating tissue at multiple wavelengths and analysing the corresponding acoustic signals. This process, known as spectral unmixing, allows for label‐free, molecular‐level tissue characterisation and adds a powerful functional dimension to structural imaging.

The interaction of light with biological tissues introduces significant challenges for spectral unmixing. Scattering and wavelength‐dependent absorption, collectively known as spectral colouring, lead to depth‐ and wavelength‐specific distortions in the detected signal [13]. These effects complicate accurate quantification of chromophore concentrations, particularly in deeper tissue regions. While traditional linear unmixing models are widely used due to their simplicity and interpretability, they assume uniform light fluence and typically perform best in superficial tissue where optical attenuation is minimal.

Recently, machine learning and deep learning approaches have emerged as promising alternatives for spectral unmixing in PAI. These data‐driven methods aim to learn the relationship between PA signals and underlying chromophore distributions directly from data, bypassing the need for explicit modelling. This flexibility makes them well suited for handling the complexity and variability of biological tissues and pathologies.

Methods such as deep‐learning‐augmented e‐multispectral optoacoustic tomography (eMSOT) [14] and superpixel‐based unmixing (SPAX) [15] have demonstrated improved accuracy and robustness in experimental and preclinical studies. This holds particularly true for complex or deeper tissues where traditional unmixing models often fall short as they cannot account for spectral colouring. These data‐driven approaches benefit from their ability to capture nonlinear relationships and adapt to heterogeneous tissue environments. However, their performance remains closely tied to the quality and diversity of training data, which is frequently limited to simulations or simplified phantoms.

To address this limitation and support continued progress, the field is placing increasing emphasis on standardisation and reproducibility. Efforts are underway to develop realistic, standardised phantoms and benchmarking datasets that better reflect the complexity of in vivo conditions. The International Photoacoustic Standardisation Consortium (IPASC) plays a key role in these developments by promoting harmonised protocols, validated reference materials and open data‐sharing initiatives [16]. These standardisation efforts are expected to strengthen validation practices and accelerate the translation of spectral PAI, particularly advanced unmixing methods, into broader research and clinical applications.

## 4 Photoacoustic Imaging of Cardiovascular Pathologies

**FIGURE 2:**
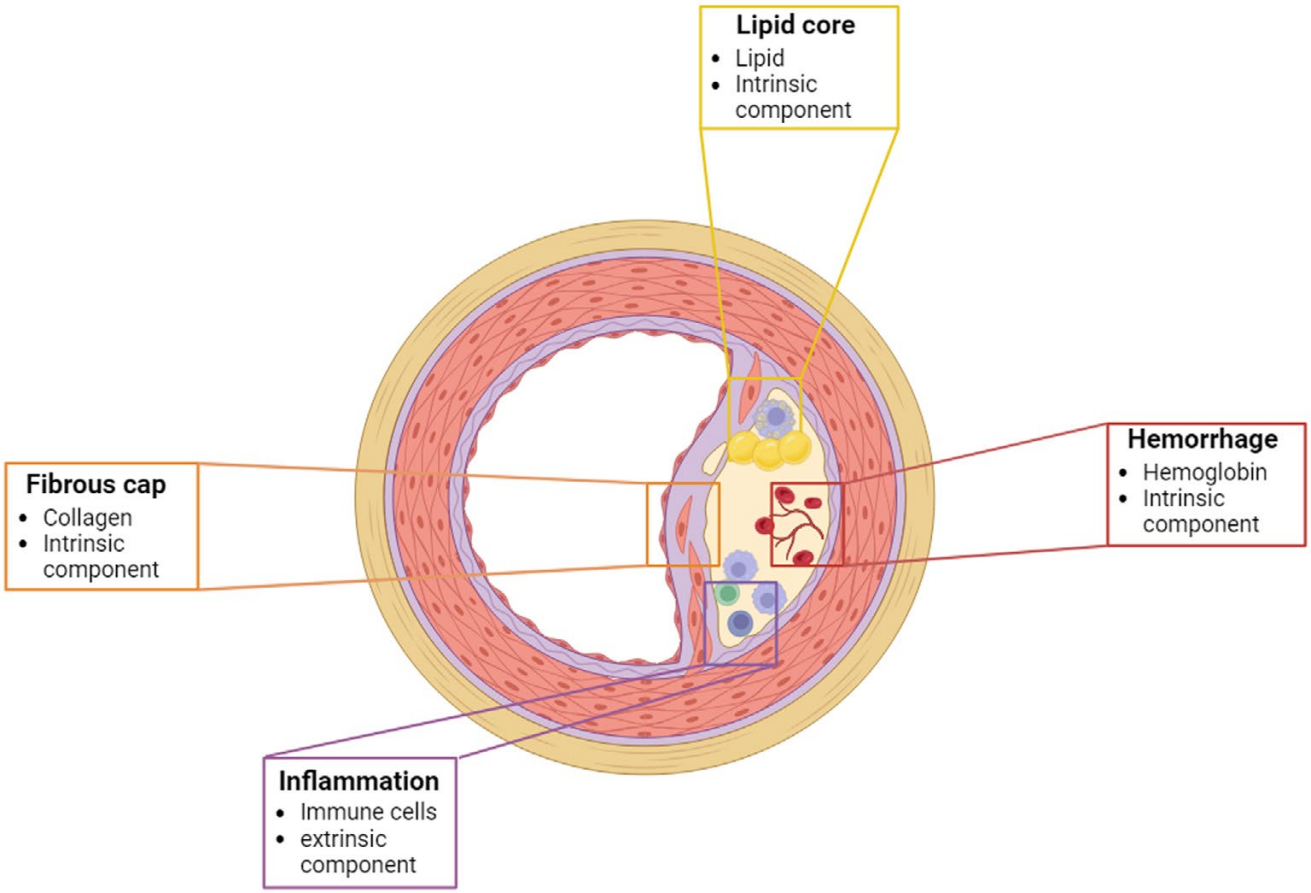
Key intrinsic and extrinsic components of atherosclerotic plaques for PAI: Lipid core, fibrous cap, intraplaque haemorrhage and inflammation.

**FIGURE 3:**
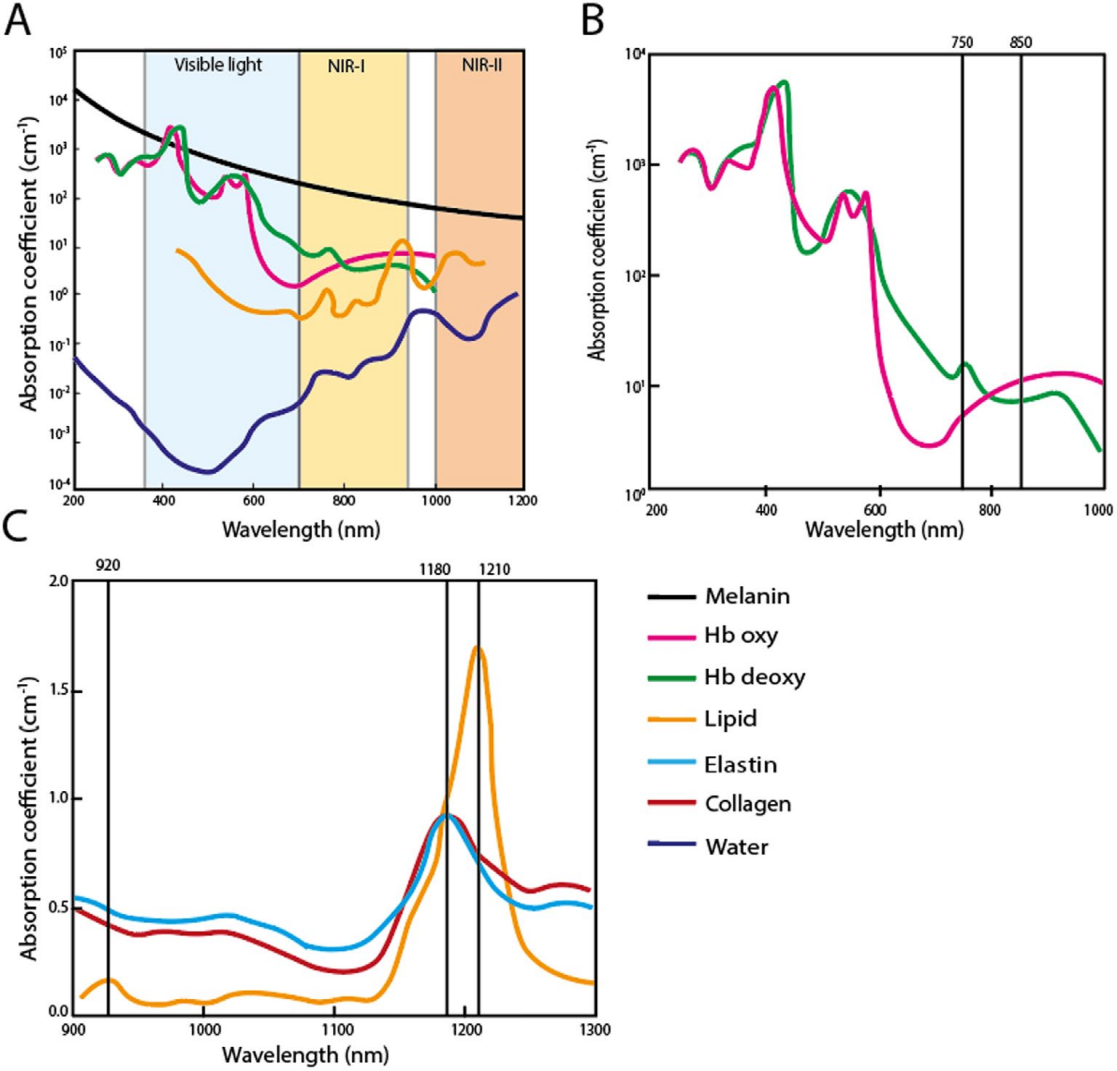
Absorption spectra of known intrinsic compounds (A) Absorption spectra of melanin, oxygenated haemoglobin (Hb oxy), deoxygenated haemoglobin (Hb deoxy), lipids and water in the visible light spectrum, NIR‐I window and NIR‐II window (B) Absorption spectra of oxygenated and deoxygenated haemoglobin, with a paired wavelength used for oximetry (C) Absorption spectra of collagen, elastin and lipid in NIR‐I and NIR‐II window.

The potential of non‐invasive PAI becomes evident when examining the complex composition and vulnerability of atherosclerotic lesions. Factors that affect plaque stability, such as lipid core (lipids), fibrous caps (collagen) and intraplaque haemorrhage (haemoglobin), have intrinsic PA properties (Figure 2). These intrinsic PA components are characterised by their strong absorbance, unique spectra and high abundance in the NIR‐I (700–900 nm) and NIR‐II (900–2500 nm and beyond) windows [4] (Figure 3A). In the following section, PAI of known intrinsic components in the context of atherosclerosis will be highlighted.

### 4.1 Lipids

Vulnerable atherosclerotic plaques are classically characterised by large lipid cores, which can constitute the majority of the plaque volume [17]. Imaging lipid accumulation in vivo holds potential to identify plaques at risk for rupture. In PAI, lipids have an absorption spectrum with three distinct peaks, around 920, 1040 and 1210 nm (Figure 3C) [18]. These three peaks correspond to the overtones of vibration of the CH2 bonds, which are abundant in lipids. Among these peaks, the 1210 nm peak has the highest intensity. Furthermore, blood absorption primarily arises from water at this wavelength and is relatively low, thereby rendering the 1210 nm peak particularly suitable for lipid imaging using PAI in the NIR‐II spectrum (Figure 3A).

Especially IVPAI, not challenged by light absorption by the skin and surrounding tissues, has been used for lipid imaging [19, 20]. Histology confirmed that lipid deposits in atherosclerotic plaques have been imaged using IVPAI in animal models and human subjects.

Recently, Sangha et al. [21] showcased that periaortic fat accumulation can be imaged in vivo using PA without an intravascular‐imaging catheter. They identified that mice on an atherosclerosis‐prone background had more periaortic fat accumulation than wild‐type mice. Karlas et al. demonstrated via non‐invasive imaging of lipids that they could distinguish carotid atherosclerosis patients from healthy volunteers, showing the potential for clinical translation.

### 4.2 Collagen

A thin, fibrous cap, comprising SMCs and extracellular matrix, is a significant characteristic of a vulnerable plaque [17]. Collagen, as the primary component of the extracellular matrix, serves as an important imaging target in atherosclerosis. The absorption spectrum of collagen has two peaks, one at 1200 nm and the other at 1450–1470 nm (Figure 3C) [22, 23]. The 1180 nm peak is instrumental since the absorption of water does not interfere, whereas at the 1450–1470 nm peak, water is the dominant absorber.

Ex vivo PAI of endarterectomy samples has successfully identified collagen‐rich areas within plaques [24]. However, in vivo imaging of collagen remains challenging. To address this, Jansen et al. [25] employed IVPAI to differentiate the adventitia, which contains connective tissue, revealing spectra consistent with theoretical elastin and collagen signatures in human atherosclerotic plaques. A major challenge is that collagen and lipids have very similar spectral profiles, making it hard to reliably distinguish between them. Advances in distinguishing lipids from collagen have also been made in other disease models, such as liver fibrosis, where both lipids and collagen are abundant, using unbiased spectral unmixing algorithms [26]. It is anticipated that further progress in in vivo imaging of collagen, a crucial component of vulnerable atherosclerotic lesions, will continue to develop.

### 4.3 Haemoglobin

Another hallmark of an unstable atherosclerotic lesion is intraplaque haemorrhage (IPH) [27, 28]. Intraplaque haemorrhage consists of erythrocytes, which contain haemoglobin. Haemoglobin has a strong and unique spectral PA signal in the NIR‐I region (Figure 3B). Upon binding with oxygen, the electron configuration of haemoglobin changes. This change shifts the PA spectrum, resulting in a different spectrum for oxygenated and deoxygenated haemoglobin.

IPH identification has been demonstrated in ex vivo human endarterectomies, confirmed by histological analysis of IPH [29]. In vivo imaging of atherosclerotic plaques is more complicated due to the imaging depth and local conditions. Muller et al. [30] identified haemorrhagic plaques in patients using IVPAI. Histological analysis confirmed the presence of IPH after the plaque was examined. Also, Karlas et al. [31] showed that besides the aforementioned lipids, haemoglobin signals could be used to detect atherosclerotic lesions.

### 4.4 Melanin

When performing PAI, it is important to consider melanin, as it can cause discrepancies in measurements for individuals with darker skin types. Although melanin is not a direct chromophore in cardiovascular imaging, it strongly affects PA signals (Figure 3A). Higher melanin concentrations, found in people with darker Fitzpatrick skin types, increase absorption at the skin surface and reduce imaging depth, leading to different results compared to lighter skin tones. Mantri et al. [32] demonstrated that this causes lower oxygen saturation readings in darker skin due to technical bias, which can be corrected using a formula based on Fitzpatrick skin types. Thus, melanin levels should be accounted for in PAI imaging to avoid bias based on skin tone when going into the clinic.

## 5 Extrinsic Photoacoustic Agents

While naturally absorbing chromophores are the basis of PA atherosclerotic lesion imaging, this is limited to components that inherently generate a strong PA signal. To overcome this limitation and enable the visualisation of specific cell types and biomarkers, extrinsic PA agents such as small molecule dyes, nanoparticles and microbubbles offer valuable opportunities by specifically targeting components with low intrinsic PA contrast.

Extrinsic PA agents offer several advantages, notably higher PA signal intensity than endogenous contrast agents, increased specificity and enhanced resolution. Additionally, PA contrast agents can be compatible with other imaging modalities, such as fluorescent imaging and MRI, facilitating multimodal imaging.

There is a broad consensus on the key characteristics needed for practical extrinsic PA components [33, 34, 35]. The ideal extrinsic agent exhibits strong absorption, low quantum yield and high molar extinction coefficient, promoting efficient PA signal generation. The agent must possess a distinct spectrum within the NIR range, allowing for signal extraction from tissue. Furthermore, these compounds' biocompatibility, stability and efficient clearance are essential for in vivo use. From a practical perspective, the ease of agent synthesis and modification is paramount, including cost‐effectiveness [33, 34, 35].

Table 1 shows examples of extrinsic PA agents, including small molecule dyes and nanoparticles, highlighting their diversity. Agents applied in atherosclerosis are included when available; otherwise, examples from other disease models demonstrate potential use. The following sections will cover key features of each category of agents, such as properties, targeting strategies and potential clinical translation, focusing on their relevance to PAI in atherosclerosis.

**TABLE 1:** Overview of photoacoustic agents.

Contrast agent	Type of contrast agent	Application	Targeting mechanism	λ_max (nm)	Disease model	Investigated in preclinical setting	Used in clinical studies	References
Indocyanine green (ICG)	Contrast dye	Various, including vasculature imaging and lymphatic system imaging	N/A	780–810	Various	In vivo	Yes^a^ [36]	[37, 38, 39]
Antibody‐indocyanine green conjugate	Contrast dye	Breast cancer imaging	Antibody to target (anti‐B7‐H3)	780–810	Tumour model	In vivo	No	[40]
Antibody‐IRDye800CW	Contrast dye	Cancer imaging, Carotid endarterectomy	Antibody to target (anti‐EGF), (anti‐VEGF‐A)	710 & 770	Tumour, atherosclerosis	No	Yes	[41, 42]
Methylene blue	Contrast dye	N/A	N/A	660–670		No	No	[43]
Alexa fluor dyes	Contrast dye	N/A	N/A	700—	N/A	No	No	[44]
Plasmonic gold nanoparticles	Gold nanoparticle	Macrophage targeting to image atherosclerotic plaques	Preferential uptake in macrophages	680	Atherosclerosis model	In vivo	No	[45]
Photoacoustic/Ultrasonic dual‐modal cRGD nanomolecular probe	Gold nanoparticle	Targeting avb3 in atherosclerotic lesions	Peptide targeting	780–790	Atherosclerosis model	In vivo	No	[46]
Targeting gold nanoshell probe	Gold nanoparticle	Imaging of atherosclerotic plaque	Antibody to target (anti‐VCAM‐1)	710/740	Atherosclerosis model	In vivo	No	[47]
Superparamagnetic iron oxide‐containing gold nanoshells	Gold nanoparticle	Tumour imaging, dual PA MRI	N/A	700–900	Tumour model	In vivo	No	[48]
ανβ3‐targeted Copper Nanoparticles	Copper nanoparticle	Angiogenesis imaging and targeting	α_ν_β_3_‐integrin antagonist—quinalone nonpeptide	770	Angiogenesis model	In vivo	No	[49]
Semiconducting polymer nanoparticles	Organic nanoparticle	anti‐CD36 semiconducting polymer to target inflammatory atherosclerotic plaques	Antibody to target (anti‐CD36)	NIR‐II	Atherosclerosis model	In vivo	No	[50]
Dendritic polyglycerol sulphate‐based	Organic nanoparticle	Imaging myocardial infarction	Binding protein motifs binding to to P‐ and L‐selectin	795	Myocardial infarction model	In vivo	No	[51]
Lipid droplet‐hitchhiking probe	Organic nanoparticle	Trojan foam cells imaging of atherosclerotic plaques	Preferential uptake in foam cells (macrophages)	795	Atherosclerosis model	In vivo	No	[52]
Porphyrin‐doped carbon nanodots	Carbon nanoparticle	Phototherapy targeting ECGR+ tumours	EGFR mediated endocytosis	686	Tumour model	In vivo	No	[53]
Functionalised carbon dot nanozymes	Carbon nanoparticle	Visual Therapy in Atherosclerosis	Peptide targeting	684	Atherosclerosis model	In vivo	No	[54]
Single‐walled carbon nanotubes (SWNTs)	Carbon nanoparticle	Selective uptake by Ly‐6Chi monocytes in the atherosclerotic plaque	Preferential uptake in monocytes	808	Atherosclerosis model	In vivo	No	[55]
Tyrosinase‐based genetic reporter	Genetically encoded	Tumour imaging	Genetic encoding	680, 750	Tumour model	In vivo	No	[56]
DrBphP‐PCM	Genetically encoded	Reporter gene	Genetic encoding	780	Tumour model	In vivo	No	[57]
NF mutants of E2 crimson	Genetically encoded	nonfluorescent E2 crimson genetic reporter	Genetic encoding	585–620	Tumour model	In vivo	No	[58]
ICG‐microbubbles	Microbubble	Targets neutrophils	Neutrophil targeting	780		In vivo	No	[59]
peptide (IMTP)‐guided nanobubbles with ICG (IMTP/ICG NBs)	Microbubble	Imaging myocardial infarction	Peptide targeting	780	Myocardial infarction model	In vivo	No	[60]
Indocyanine green‐doped targeted microbubbles	Microbubble	evaluation of coronary microvascular dysfunction	Protein targeting (fibrin)	780/803	Conorary microvascular dysfunction model	In vivo	No	[61]
pH‐sensitive pHLIP (V7)conjugated to 750 NIR fluorescent dye	Activatable	targeted probe for pancreatic adenocarcinoma	pH sensing	750	Tumour model	In vivo	No	[62]
pH‐responsive near‐infrared (NIR) croconine (Croc) dye	Activatable	Tumour sensing	pH sensing	680, 810	Tumour model	In vivo	No	[63]
semiconducting oligomer with amplified brightness and pH‐sensing capability	Activatable	Tumour sensing	pH sensing	685	Tumour model	In vivo	No	[64]
Dual‐activated photoacoustic probe for reliably detecting hydroxyl radical	Activatable	Detecting Hydroxyl radical in ischemic cardiovascular disease	ROS sensing	680–880	Myocardial infarction model	In vivo	Testing in human samples	[65]
Reactive oxygen species‐responsive nanoparticle	Activatable	ROS sensing for atherosclerosis theranostics	ROS sensing	1064	Atherosclerosis model	In vivo	No	[66]

*Note:* Types of contrast agents include: contrast dyes, nanoparticles (gold/metal, organic, carbon), genetically encoded, microbubbles and activatable.

^a^ Also approved for clinical use.

### 5.1 Small Molecule Dyes

Small molecule dyes are highly attractive agents for PAI. Small molecule dyes are low molecular weight organic compounds that typically have well‐defined chemical structures. Small molecule dyes are used due to their strong light‐absorbing properties. Most dyes have low toxicity and good biocompatibility [33]. Moreover, small molecule dyes can be conjugated to targeting agents such as antibodies, peptides or aptamers to enable selective imaging of molecular markers.

One of the most prevalent PA dyes is Indocyanine green (ICG). ICG has a high affinity for albumin and is, among others, used in the clinic as an FDA‐approved blood flow contrast agent [67]. ICG has both PA properties, with a peak at 810 nm and fluorescent properties. Many clinical studies have been performed with ICG, as the fluorescent properties have made it a useful tool for fluorescence‐guided surgery [68, 69].

Utilising ICG in combination with PAI, Jonas et al. [70] showed the therapeutic effects of compounds on lymphatic drainage. It is also used to image vascular permeability in tumours [37]. In this latter study, the PA signal could distinguish between control and treatment groups before tumour growth inhibition was detected using conventional methods. ICG has also been used in clinical trials with PAI to image blood flow [38].

Additionally, ICG has been conjugated to antibodies to target B7‐H3, a breast cancer‐associated molecular target [71]. Using this system, they could accurately distinguish between breast cancer and normal breast tissue in mice. The ease of ICG conjugation to targeted antibodies provides a versatile PA platform.

Similarly to ICG, IRDye800CW (also referred to as CW800) has been explored for photoacoustic imaging applications, particularly when conjugated to antibodies. For instance, CW800 has been conjugated to anti‐epidermal growth factor receptor (EGFR) antibodies for the detection of occult lymph node metastases in humans, demonstrating its potential for molecularly targeted imaging [41, 42]. This approach may also hold relevance for atherosclerosis, as EGFR is involved in inflammatory, oxidative and proliferative signalling, all implicated in vascular pathophysiology.

Steinkamp et al. [72] used MSOT and CW800 conjugated to anti‐VEGF‐A to assess plaque vulnerability in human carotid lesions. They demonstrated the safety and feasibility of molecular assessment in vivo although the used dose did not show sufficient target‐specific contrast.

### 5.2 Nanoparticles

Nanoparticles (NPs) are engineered particles, typically 1 to 100 nm in size, that serve as contrast agents due to their strong optical absorption and efficient conversion of light into acoustic signals [73]. NPs can circulate in biological systems and accumulate at target sites, enhancing imaging sensitivity and specificity. They are composed of a diverse scala of materials, including metal‐based NPs, carbon‐based NPs and organic‐based NPs. While a detailed discussion of their physics, modifications and numerous variations is outside the scope of this review, we will highlight the most relevant examples for cardiovascular research.

Among the metal‐based NPs, gold NPs are widely recognised for their relative inertness, stability, biocompatibility and targeting capabilities. Different geometries and the PA properties of gold NPs are extensively reviewed elsewhere [74, 75]. Gold nanoshells targeting VCAM‐1, a known marker of early endothelial cell activation, have been developed [47]. It was shown ex vivo and in vivo that the targeted nanoshells accumulate in atherosclerotic plaques in the heart and aorta, allowing for imaging of vulnerable plaques.

Another class of nanoparticles is carbon nanoparticles (CNPs), which allow both covalent and noncovalent modifications. Dyes, drugs or biomolecules can be conjugated to CNPs, allowing for the addition of targeting and signalling molecules [76, 77]. Bio‐compatibility studies for the in vivo use of carbon nanoparticles (CNPs) are still underway. These investigations are challenging because CNPs can vary widely in their size, shape, surface chemistry and functionalisation, all of which influence how they interact with biological systems [78]. Particularly interesting for cardiovascular disease is the use of carbon nanotubes as described by Gifani et al. They demonstrated that carbon nanotubes are selectively taken up by inflammatory monocytes and foamy macrophages, resulting in inflammation‐specific imaging of murine atherosclerotic plaques [79].

Organic‐based nanoparticles are also a considerable group with a high base material diversity. These organic NPs can function as direct signalling compounds, enhance other signalling compounds or encapsulate other compounds to improve biocompatibility. Most often used are polypyrrole NPs and porphyrin NPs, which are extensively reviewed elsewhere [80]. An exciting application of organic‐based NPs is lipid NPs used to target foam cells in atherosclerosis, creating ‘Trojan foam cells’. For this, lipid‐rich cells at plaque locations are targeted by inducing lipid droplets in immune cells with specially designed liposomes. The liposomes carry a lipid‐based probe that produces strong fluorescence and PA signals, allowing detailed imaging of plaque areas [52].

The field of NPs with PA properties is rapidly evolving and offers numerous possibilities. Yet, standardisation within this field remains lacking, particularly concerning biocompatibility and related testing. Substantial efforts should be directed at refining biocompatibility assessments to progress toward applications in clinical settings.

### 5.3 Activatable Photoacoustic Agents

Activatable PA agents produce PA signals depending on their environmental cues. Considerable diversity exists in their composition and the stimuli they detect (Table 1). A notable category of activatable PA probes is those that react to reactive oxygen species (ROS). ROS are produced in highly inflammatory tissues and can be used as a marker of instability in atherosclerotic plaques [81]. For example, Weber et al. [33] demonstrated a modified heptamethine dye, which exhibits a redshift in absorption spectrum when encountering hydrogen peroxide, allowing for visualisation of pathophysiological concentrations of this ROS. Furthermore, gold‐based nanoparticles have been used to detect ROS [82]. Other activatable PA agents change when encountering MMPs [83] or respond to pH [62, 63, 64]. Similar to NPs, activatable PA agents represent a highly diverse class of contrast agents. However, the field currently lacks standardisation and sufficient comparative data, rendering it difficult to assess its translational potential.

### 5.4 Genetically Encoded Tags

An intermediate between endogenous and extrinsic PA agents involves genetically encoded tags. These genetically encoded tags utilise reporter genes that produce a PA contrast agent under the control of a promoter of the gene of interest. Genetically encoded green fluorescent proteins (GFPs) are a well‐known example of this principle. GFP cannot be used as the wavelength is unsuitable for PAI and has a low quantum yield. Modifications to the GFP‐like molecules result in proteins that absorb wavelengths within the NIR range and are more suitable for PA [58]. An alternative approach exploits the intrinsic PA properties of melanin. Paproski et al. demonstrated the use of a tetracycline‐inducible melanin expression system to detect tumours expressing tyrosinase. This approach enabled a sharp distinction between tyrosinase‐positive and tyrosinase‐negative tumours [56]. In the context of atherosclerosis, genetically encoded tags have not yet been applied but hold promise for translational and discovery‐driven research due to the high specificity of these tags.

### 5.5 Microbubbles

Microbubbles are contrast agents traditionally used for US imaging [84]. A microbubble consists of a gaseous core encapsulated by a shell of proteins, lipids or polymers. The gas core causes the microbubble to be highly echogenic, which is ideal for US imaging. As most PAI is combined with US, a logical progression is to use microbubbles for PAI as well. Nanoparticles or PA dyes can be combined with microbubbles to work synergistically. For example, Awen et al. [61], showed ICG microbubbles targeting fibrin to detect microthrombi associated with atherosclerotic lesions. While their study employed optical fluorescence imaging, the potential translation of this approach to PAI is promising. PA microbubbles are particularly attractive for clinical translation, as they build upon microbubble formulations already approved for diagnostic US. Moreover, ICG is FDA‐approved, further supporting translational feasibility. Nonetheless, the addition of PA components can influence microbubble stability, circulation time and biodistribution, and these parameters require careful optimisation during development.

## 6 Concluding Remarks

PAI is a newly emerging imaging modality with enormous potential in preclinical translational research and future clinical imaging. The imaging modality offers superior depth and resolution compared to other modalities. Ongoing technological advancements will further enhance these qualities, particularly for clinical applications. PAI of intrinsic components, especially haemoglobin, lipids and collagen, has demonstrated its effectiveness in assessing cardiovascular pathologies. Additionally, extrinsic contrast agents, such as dyes and nanoparticles, allow imaging of non‐intrinsic components. Together, these capabilities position PAI as a versatile and powerful tool for advancing cardiovascular disease research and paving the way toward clinical translation.

## Author Contributions

**Kayleigh van Dijk:** conceptualization (equal), writing – original draft (lead), writing – review and editing (lead). **Margreet R. de Vries:** conceptualization (equal), funding acquisition (lead), supervision (lead), writing – original draft (supporting), writing – review and editing (supporting).

## Conflicts of Interest

The authors declare no conflicts of interest.

## Data Availability

The data that support the findings of this study are available from the corresponding author upon reasonable request.
